# Notes

**Published:** 1903-04

**Authors:** 


### NOTES.

Dog is Man's Best Friend. In a Missouri town, says the *Atchison* (Kan.) *Globe*, a man brought suit for $200, claiming a neighbor had killed his dog, and he engaged Senator Vest to plead his case. The Senator made the following speech:

"Gentlemen of the Jury: The best friend a man has in the world may turn against him and become his enemy. His son or daughter that he has reared with loving care may prove ungrateful. Those who are nearest and dearest to us, those whom we trust with our happiness and our good name may become traitors to their faith. The money that a man has he may lose. It flies away from him, perhaps, when he needs it most. A man's reputation may be sacrificed in a moment of ill-considered action. The people who are prone to fall on their knees to do us honor when success is with us may be the first to throw the stone of malice when failure settles its cloud upon our heads. The one absolutely unselfish friend that a man can have in this selfish world, the one that never deceives him, the one that never proves ungrateful and treacherous, is his dog.

"A man's dog stands by him in prosperity and in poverty, in health and in sickness. He will sleep on the cold ground, where the wintry wind blows and the snow drives fiercely, if only he may be near his master's side. He will kiss the hand that has no food to offer. He will lick the wounds and sores that come in encounter with the roughness of the world. He guards the sleep of his pauper master as if he were a prince. When all other friends desert he remains. When riches take wings and reputation falls to pieces he is as constant in his love as the sun in its journeys through the heavens. If fortune drives the master forth an outcast in the world, friendless and homeless, the faithful dog asks no higher privilege than that of accompanying, to guard against danger, to fight against his enemies, and when the last scene of all comes, and when death takes the master in its embrace and his body is laid away in the cold ground, no matter if all other friends pursue their way, there by the graveside may the noble dog be found, his head between his paws, his eyes sad but open in alert watchfulness faithful and true even in death."

Then Mr. Vest sat down. He had spoken in a low voice, without a gesture. He made no reference to the evidence or the merits of the case. When he finished the judge and the jury were wiping their eyes. The jury filed out, but soon returned with a verdict in favor of the plaintiff for $500. He had sued for $200. It is even said that some of the jurors wanted to hang the defendant.

A Refuge for Stray Animals. A refuge for friendless dogs and cats has been established by the Misses Ritter at their pleasant home in Mount Auburn, near Cincinnati. Neighbors say that no dog or cat was ever turned away from the door. It is the residence of Miss Cecilia Ritter and her sister. Both women have long been known for their humanity toward dumb animals, but Miss Cecilia Ritter has been especially solicitous for their welfare. No stray dog or cat escapes her attention. If they should happen to be injured her dress is never too fine to prevent her stooping and raising them to her arms. Then carefully secreting her charge under her fur cloak, she carries it away to her home, where every effort is made to nurse it back to health. If the cat or dog should be injured beyond hope of being afforded relief, then the two sisters gently carry it to a box on the premises, where it may be asphyxiated with chloroform. Hundreds of crippled animals and fowls have been put away in this manner. In fact, every girl and boy in the neighborhood of the Ritter home is an especially commissioned individual to bring in stray dogs and cats.—*Ohio State Journal.*

Tuberculosis Serum Has Been Discovered. Dr. Mavmorek, the bacteriologist of the Pasteur Institute in Paris has succeeded after years of experiments in securing tuberculosis serum which in numerous cases has proved a specific cure. Dr. Mavmorek will soon present the results of his investigation to the Paris Academy of Science.

The discovery was completed nine months ago, but Dr. Mavmorek hesitated to make it public because he feared the demand for serum would have been so great that he would have been unable to satisfy it at that time.

Germ of Rabies Found (?) According to *Il Secolo*, of Milan, an Italian scientist has discovered the microbes of hydrophobia. At a recent sitting of the Medical Society, Professor Sormagni, of the University of Pavia, announced that after a long series of experiments he had at last succeeded in isolating the microbe in question.

It is a well-known fact that Dr. Pasteur's treatment of the disease has not been controlled with absolute scientific certainty, owing to the fact that the bacteria producing the disease have not been sufficiently identified.

Dr. Racolli, the highest medical authority in Rome, says that if Profesor Sormagni's claim can be proved there is no reason why the treatment of hydrophobia should not become revolutionized and take its place among the few exact sciences of materia medica.

Hospital for Birds. Although New York has many shops for the sale of birds, their belongings and requirements, only recently has it possessed a hospital for the reception of feathered sufferers. So far as is known, also, this is the only institution of this kind in existence anywhere, according to the New York *Commercial Advertiser.* Miss Virginia Pope, the proprietress and head physician, is an enthusiastic lover of birds. She has made their anatomy, nature, habits, idiosyncrasies, and ailments the study of her life, even going to the Black Forest and remaining there for a considerable period in order to thoroughly acquaint herself with canaries. She not only goes out to visit sick birds professionally at their owners' residences, but maintains this hospital and sanitarium, where she receives feathered patients and ministers to their woes with the gentle and skilled attention of a trained enthusiast, and she also takes in as boarders pet birds whose owners are compelled, through absence from the city or some other reason, to part with them temporarily.

The establishment consists of several rooms. The largest is that allotted to the boarders of the bird community. It is a triangular-shaped one of considerable size, the base of the triangle facing the street, and being almost all window, with a southern exposure. The bright sunshine came pouring in, and the floods of melody issuing from more than one hundred fluffy little throats made it seem a very happy place when the reporter called a day or so ago. All along the walls hung cages with small occupants—canaries, thrushes, orioles, etc., generally one in each cage. Near the window stood an aviary containing a happy family of about a dozen birds of different species—goldfinches, bullfinches, linnets, and Java sparrows—all living in amity.

There were also several paroquets, a splendid pair of cardinals, an inquisitive-looking robin, and a soft-voiced parrot. Although this was the boarders' room, some of the birds in it were former patients that, having recovered from the ailment or operation which necessitated their sojourn with the doctor, were now advanced convalescents, soon to be restored to their rejoicing owners. In the inner rooms were the little bipeds under medical treatment. Birds are subject to many of the ailments to which human flesh is heir. They have, according to the doctor, rheumatism, dyspepsia, asthma, consumption, palpitation of the heart, fevers, paralysis, and even corns. Some of the patients were in bed. The bed is a little pasteboard box; the mattresses are made of soft old linen damask, and the bird is tucked in carefully and comfortably, only his little round head and bright eyes being visible. Asthma is the form of disease to which birds are most liable, and most of the patients here suffer from this trouble. Asthmatic patients are treated by having a hot water-bag placed on top of their cages and a flannel cloth thrown over all—a small space being left for breathing air to enter. Sometimes, too, the bird is made to lie on a flannel-swathed hot water bag.

Heat is the most important factor in the medical treatment of birds. The paraphernalia in evidence here for the alleviation of bird illness is quite a formidable one. Besides the hot water bags and thermometers, bandages, rubber gloves, bird-sprinklers, medicated cotton, and bottles of all kinds of medicine and cocaine are visible. All these enter into the healing processes carried on at the sanitarium. Both allopathic and homœopathic remedies are resorted to, according to the nature of the ailment. Massage also is found to be very beneficial in some cases of bird trouble.

A very powerful microscope was at hand—an instrument frequently used in the examination of patients. Not only sufferers from diseases are treated here—victims of accident also are often brought and successfully treated by the doctor, who is a skilled bird surgeon. A bird with a broken leg has it set and bandaged, after which it is put in the bandage swing, formed of a strap of strong linen with two slits cut in it. The bird's legs are inserted in the slits, and he is left to swing until his recovery. Mites are a pest to which canaries are at times liable. They are tiny red insects which prey upon the little victim until his life is sapped away unless something is done to relieve him. If a flannel cloth be placed at night on the cage the mites will be found in the morning to have left the bird for the warm wool. If this is repeated night after night and the cage thoroughly cleansed—kerosene is valuable for this—in time the bird will be rid of the pests.

---

In two green horses shipped from the West and suffering with laryngitis as a complication of influenza, followed by a persistent cough, spasmodic in character, the use of glyco-heroin gave prompt relief and early recovery from the same in the practice of Dr. W. Horace Hoskins.

